# Eucommia Polysaccharides Ameliorate Aging-Associated Gut Dysbiosis: A Potential Mechanism for Life Extension in Drosophila

**DOI:** 10.3390/ijms24065881

**Published:** 2023-03-20

**Authors:** Jing-Jing Wei, Xiu-Juan Li, Wei Liu, Xue-Jun Chai, Xiao-Yan Zhu, Peng-Hao Sun, Feng Liu, Yong-Kang Zhao, Jun-Lang Huang, Ya-Fei Liu, Shan-Ting Zhao

**Affiliations:** 1 College of Veterinary Medicine, Northwest A&F University, Xianyang 712100, Chinalxj2493435@163.com (X.-J.L.);; 2 College of Basic Medicine, Xi’an Medical University, Xi’an 710068, China

**Keywords:** Eucommiae polysaccharides (EPs), life span, aged, microbiome remodeling, reactive oxygen species (ROS)

## Abstract

The gut microbiota is increasingly considered to play a key role in human immunity and health. The aging process alters the microbiota composition, which is associated with inflammation, reactive oxygen species (ROS), decreased tissue function, and increased susceptibility to age-related diseases. It has been demonstrated that plant polysaccharides have beneficial effects on the gut microbiota, particularly in reducing pathogenic bacteria abundance and increasing beneficial bacteria populations. However, there is limited evidence of the effect of plant polysaccharides on age-related gut microbiota dysbiosis and ROS accumulation during the aging process. To explore the effect of Eucommiae polysaccharides (EPs) on age-related gut microbiota dysbiosis and ROS accumulation during the aging process of Drosophila, a series of behavioral and life span assays of Drosophila with the same genetic background in standard medium and a medium supplemented with EPs were performed. Next, the gut microbiota composition and protein composition of Drosophila in standard medium and the medium supplemented with EPs were detected using 16S rRNA gene sequencing analysis and quantitative proteomic analysis. Here, we show that supplementation of Eucommiae polysaccharides (EPs) during development leads to the life span extension of Drosophila. Furthermore, EPs decreased age-related ROS accumulation and suppressed *Gluconobacter*, *Providencia*, and *Enterobacteriaceae* in aged Drosophila. Increased *Gluconobacter*, *Providencia*, and *Enterobacteriaceae* in the indigenous microbiota might induce age-related gut dysfunction in Drosophila and shortens their life span. Our study demonstrates that EPs can be used as prebiotic agents to prevent aging-associated gut dysbiosis and reactive oxidative stress.

## 1. Introduction

Aging is a gradual and complex process characterized by the progressive degeneration of cells, tissues, and organs, making individuals sensitive to diverse stimuli, such as cytokines and ROS, and more likely to suffer from chronic and debilitating diseases [1,2]. Particularly vulnerable are tissues of the gastrointestinal epithelial barrier, as it is in constant exposure to environmental insults and therefore undergoes rapid cellular turnover to maintain barrier integrity [3]. The gut microbiota affects host health and play key roles in the development and homeostasis of the host immune system and gut epithelial barrier [4]. Compared with primates and rodents, Drosophila contain only a few numbers of bacteria species in the gut lumen (fewer than 30 species), with *Actinobacteria, Bacteroidetes, Firmicutes*, and *Proteobacteria* as the predominant phyla [5]. Opportunistic pathogens, such as *Providencia, Enterobacteria, Gluconobacter, Serratia, Erwinia, Enterococci*, and *Pseudomonas*, are also detected [5,6]. 

During aging, the gut microbiota undergoes changes in the community structure and abundance, shows higher variability in the proportions of different taxa, and altered diversity [1]. These changes can affect the immunity and life span of the host. Thus, multiple interventions for optimizing the microbial flora profile have been proven to be beneficial in extending life spans in various animal models. Although great advances have been made in understanding the causes and mechanisms of senescence, antiaging interventions for efficiently improving the health span and reducing the risk of age-related diseases are still urgently needed. 

Eucommiae cortex, the bark of Eucommia ulmoides Oliver, is known for its “medicine food homology” in China. Recently, 204 compounds have been isolated from Eucommiae cortex using advanced techniques. These compounds belong to seven categories, including EPs, which have wide pharmacological effects and clinical applications, such as antioxidant, antifatigue, and immunomodulatory effects [7,8,9,10]. Recent studies have reported that polysaccharides could bring beneficial effects, such as reducing pathogens and increasing favorable bacteria [11]. However, polysaccharides from various sources present distinct structural types and differently influence the gut microbiota [12]. It remains unclear whether EPs affect the gut microbiota of Drosophila and improve its health span. 

Drosophila melanogaster is one of the best-described model organisms for studying development and aging. In this study, we investigated the effects of EPs on the life span of Drosophila melanogaster. Our results demonstrated that supplementation of EPs extends the life span of Drosophila and reduces age-related oxidative stress and gut dysbiosis. In addition, *Gluconobacter*, *Providencia*, and *Enterobacteriaceae* might act as potential targets for relieving the symptoms of aging, and EPs might be used to prevent age-related symptoms and disease.

## 2. Results

### 2.1. Morphology, Monosaccharide Composition, and Molecular Weight of EPs

**Figure 1:**
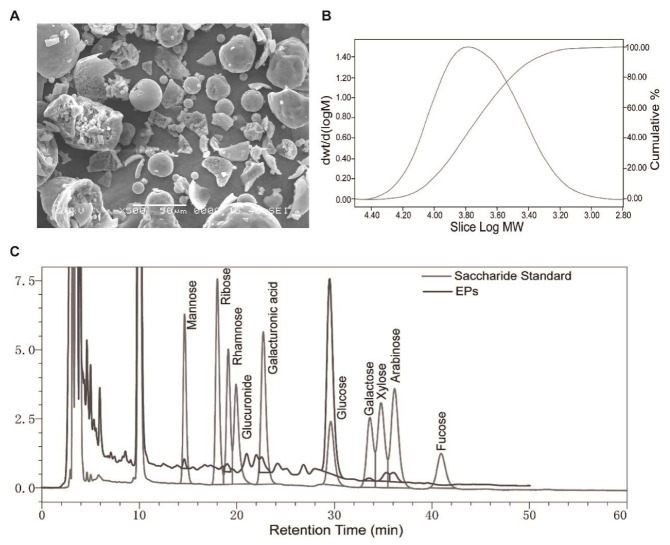
Morphology, monosaccharide composition, and molecular weight of EPs. (**A**) Scanning electron microscopy image of EPs. (**B**) Molecular weight distribution of EPs. (**C**) Elution profiles of the saccharide standard and EPs using high-performance liquid chromatography.

An electron microscopy image of EPs is shown in Figure 1A. The main monosaccharide composition of EPs is shown in Figure 1C and Table 1. The molecular weight of purified EPs ranged from 630 to 251,000 Da (Figure 1B). 

### 2.2. Supplementation of EPs with a Proper Concentration Extended the Life Span of Drosophila

**Figure 2:**
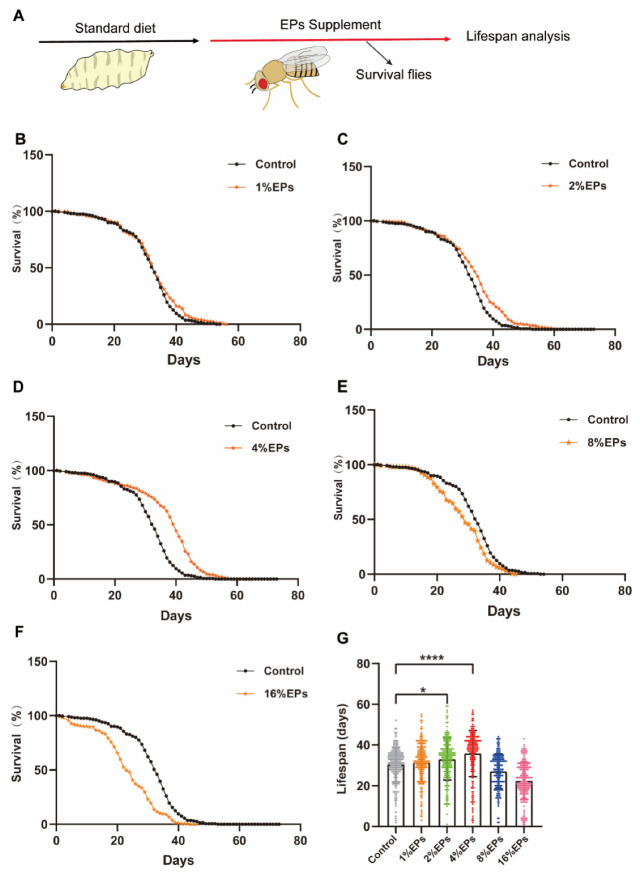
EPs with proper concentrations promoted the survival and extended the life span of Drosophila. (**A**) Schematic diagram of the experiment. (**B**–**F**) Percentage survival of flies in the control group fed with standard medium and groups supplemented with 1%, 2%, 4%, 8%, and 16% EPs. (**G**) Life span of flies in the control group and EP groups with different concentrations. Mean ± SEM (*n* = 200). The details of the statistical tests used in this and all subsequent figures are given in the Section 4. * *p* < 0.05, **** *p* < 0.0001.

To explore the effect of EPs on the life span of Drosophila melanogaster, newly ecolsed flies were fed with a medium containing 1%, 2%, 4%, 8%, or 16% EPs throughout their whole lives and surviving flies were counted every day (Figure 2A). Our results indicated that a low concentration of EPs (1%) did not show any significant difference compared to the control group (*p* > 0.05); see Figure 2B,G and Table 2; however, both 2% and 4% EPs significantly extended the life span of flies (*p* < 0.05); see Figure 2C,D,G and Table 2. In contrast, 8% and 16% EPs showed negative effects (*p* < 0.05); see Figure 2E–G and Table 2.

### 2.3. Supplementation of EPs Promotes Locomotor Activity and Fecundity in Old Flies

**Figure 3:**
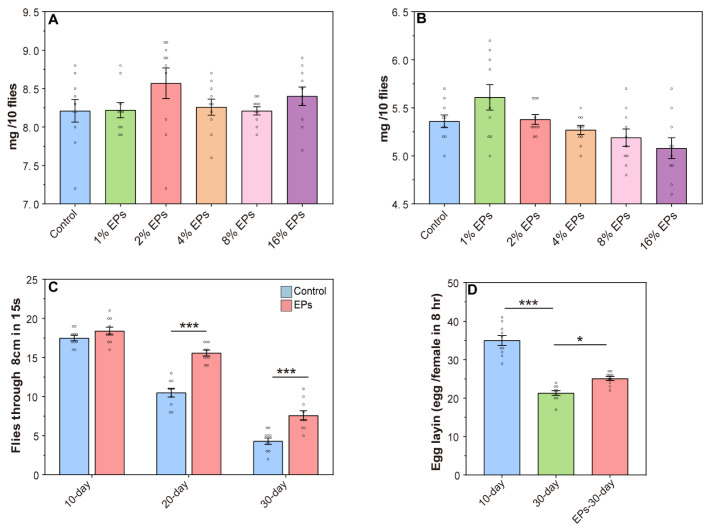
Effect of EPs on the behavior of aged flies. (**A**) Effect of EP (1–16%) supplementation on the weights of female flies. (**B**) Effect of EP (1–16%) supplementation on the weights of male flies. (**C**) Effect of EPs on the locomotor activity of old flies (**D**) Effect of EPs on the fecundity of old flies. * *p* < 0.05, *** *p* < 0.001.

For safety evaluation, the body weight of the flies was assessed (10 flies per sample). As shown in Figure 3A,B, compared to the control group, the EP groups showed no significant change in body weight. Based on this evidence, 4% was chosen as the optimal concentration of EPs for further experiments. 

One hallmark of aging in many species, including Drosophila melanogaster, is the decline in locomotor activity. In the control group, we observed a steady decline in climbing activity from 10 days to 30 days, and EP supplementation has no significant effect on climbing activity in young flies (10 days old), while we observed a significant increase in climbing activity during aging (20 and 30 days old) with EP supplementation (Figure 3C). In females, fecundity is another index to evaluate aging, and we observed increased fecundity in aging with EP supplementation (Figure 3D). These results further indicated that supplementation with EPs at a proper concentration during development delays aging and promotes longevity. 

### 2.4. EPs Reduced the Oxidative Stress of Aged Drosophila

**Figure 4:**
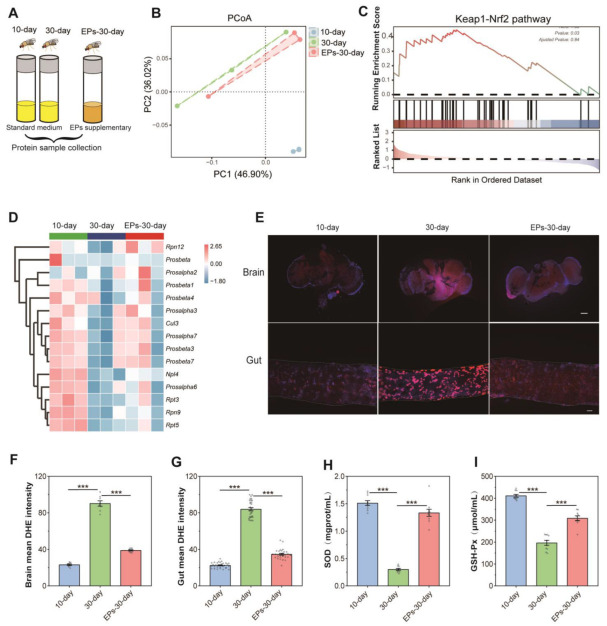
EPs inhibited oxidative stress in aged Drosophila. (**A**) Schematic diagram of the proteomics procedure (10-day- and 30-day-old flies in the control group and 30-day-old flies in the EP groups, 25 males and 25 females per sample, *n* = 3). (**B**) PCoA of the proteomics of 10-day- and 30-day-old flies in the control group and 30-day-old flies in the EP groups. (**C**) GSEA plot depicting the enrichment proteins involved in Keap1–Nrf2 signaling (NES = 1.33, *p* = 0.03). Red: aged (positively correlated), Blue (negatively correlated). (**D**) Heatmap of the enrichment proteins involved in Keap1–Nrf2 signaling. (**E**) DHE staining in the brains (upper) and guts (below) of 10-day- and 30-day-old flies in the control group and 30-day-old flies in the EP groups (bar = 100 μm). (**F**) DHE intensity of the brains of 10-day- and 30-day-old flies in the control group and 30-day-old flies in the EP groups. (**G**) DHE intensity of the guts of 10-day- and 30-day-old flies in the control group and 30-day-old flies in the EP groups. (**H**) Activity of T-SOD in 10-day- and 30-day-old flies in the control group and 30-day-old flies in the EP groups. (**I**) Activity of GSH-Px in 10-day- and 30-day-old flies in the control group and 30-day-old flies in the EP groups. *** *p* < 0.001.

Protein samples from flies at the 10th day, in the control group at the 30th day, and flies in the EPs groups at the 30th day were collected, and quantitative proteomics assays were performed (Figure 4A). We computed fold-changes (FCs) between 10-day-old and 30-day-old flies to determine differential protein expression. A biologically significant FC of ≥1.5 and ≤0.5 was kept for upregulated and downregulated proteins, respectively. In addition, A *p*-value of ≤0.05 was applied for statistical significance [13]. In total, 1032 nonredundant proteins were identified and quantified using MaxQuant 2.0.3.0, while 998 proteins were screened out (unique peptide ≥ 2). Our results indicated that the PCoA of proteomic data showed a clear difference between the 10-day-old and the 30-day-old flies, while the data of the 30-day-old flies fed with EPs were more close to those of the 10-day-old flies in the control group (Figure 4B).

For sequencing data, gene set enrichment analysis (GSEA) is used to analyze enrichment without a threshold. There is no doubt that it is the most effective method for identifying gene sets with differential expression levels among groups. We used GSEA to evaluate the differential expression protein levels between 10-day- and 30-day-old flies in the control group and 30-day-old flies in the EP groups. Our results showed that many gene sets related to the Kelch-like ECH-associated protein 1 (Keap1)–nuclear factor erythroid 2-related factor 2 (Nrf2) pathway of the 30-day-old flies were significantly downregulated compared to the 10-day-old flies in the control group, while they were upregulated in 30-day-old flies fed EPs (Figure 4C,D). These data revealed that EP supplementation increases the antioxidant-related Keap1–Nrf1 pathway in aged flies.

ROS-mediated redox signaling is implicated in a wide range of biological phenomena associated with aging, including inflammation, senescence, and dysregulation of biochemical pathways [14]. To explore the antioxidant effect of EPs, we examined the ROS levels in the brain and gut using Dihydroethidium (DHE)staining, which is well known as the most sensitive and specific probe to detect superoxide radicals in living tissues and it intercalates into DNA and displays a red fluorescence signal [15]. As shown in our results, remarkable ROS accumulation was found in the brain and gut of 30-day-old flies compared with 10-day-old flies in the control group. However, the level of ROS decreased in the brain and gut of 30-day-old flies in the EP groups compared with the control group (Figure 4E–G, *p* < 0.001). The activity of SOD and GSH-Px is a crucial enzyme-driven antioxidant defense system in organisms that scavenges free radicals to maintain redox homeostasis in cells [16]. Thus, the activity of SOD and GSH-Px was detected using commercially available kits. Our results showed that in the control group, the activity of T-SOD and GSH-Px dramatically decreased in 30-day-old flies compared with 10-day-old flies. Interestingly, the activity of T-SOD (Figure 4H) and GSH-Px (Figure 4I) significantly increased in 30-day-old flies in the EP groups compared with 30-day-flies in the control group. These results indicate that EPs reduce age-related oxidative stress in Drosophila.

### 2.5. EPs Selectively Suppressed Gluconobacter, Providencia, and Enterobacteriaceae, the Main Bacterial Source of ROS from the Microbiome in Aged Drosophila

**Figure 5:**
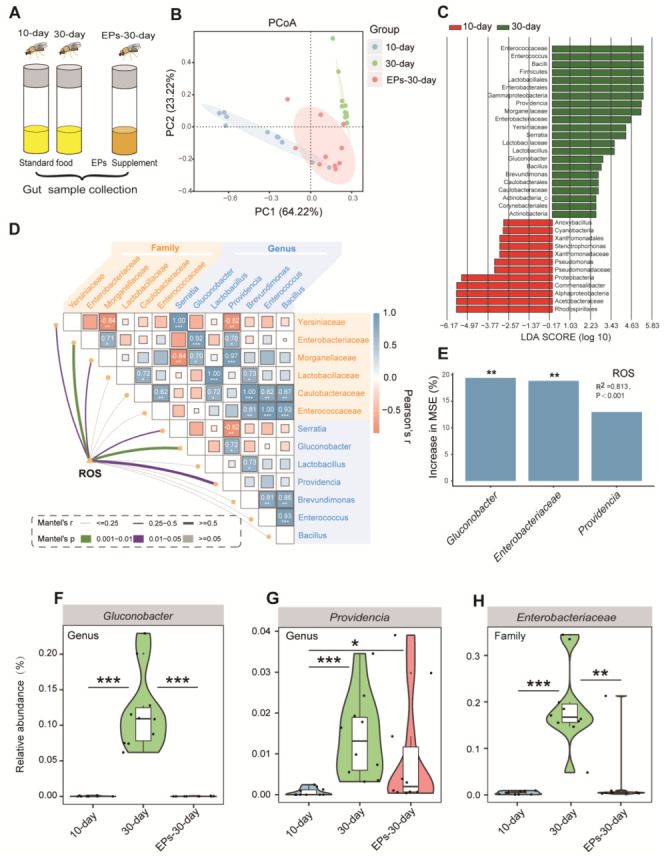
EPs remodeled the gut microbiota of aged Drosophila. (**A**) Schematic diagram of the experimental procedure (10-day- and 30-day-old flies in the control group and 30-day-old flies in the EP groups, 15 males and 15 females per sample, *n* = 10). (**B**) ASV-based PCoA with the Bray–Curtis distance (for principal component 1 and principal component 2), showing the variations of the gut microbial beta diversity of 10-day- and 30-day-old flies in the control group and 30-day-old flies in the EP groups and assessed with permutational multivariate analysis of variance (PERMANOVA). (**C**) Discriminative taxa between the 10-day- and 30-day-old flies (log_10_ LDA > 2) determined by LEfSe. (**D**) Correlations between ROS accumulation and microbial diversity of flies are shown, with a color gradient denoting Spearman’s correlation coefficient. The ROS level was related to microbial diversity using partial Mantel tests. The edge width corresponds to the Mantel’s r statistic for the corresponding distance correlations, and the edge color denotes the statistical significance based on 999 permutations. (**E**) RF mean predictor importance (percentage of increase in mean square error) of dominant phyla (>5% of total community) as drivers for ROS accumulation in old flies. Percentage increases in the MSE (mean square error) of variables were used to estimate the importance of these predictors, and higher MSE% values imply more important predictors. (**F**–**H**) Comparison of the proportions of *Gluconobacter* (**F**), *Providencia* (**G**), and *Enterobacteriaceae* (**H**) in the gut microbiota detected using 16S sequencing analysis. Significance levels are as follows: * *p* < 0.05, ** *p* < 0.01, *** *p* < 0.001.

It was previously demonstrated that EPs can improve cognitive and social behavior through the actions of the gut microbiota [17]. We wondered whether EPs could affect the gut microbiota in Drosophila. We collected guts of 10-day- and 30-day-old flies in the control group and 30-day-old flies in the EP groups (Figure 5A), and assessed the microbial composition and diversity using 16S rRNA amplicon sequencing. Notably, we found remarkable differences in beta diversity between the 10-day- and 30-day-old flies in the control group; however, the microbiota composition of the 30-day-old flies in the EP groups rescued the age-related disturbance in the gut microbial community to some extent (Figure 5B). 

For further investigation, all effective sequences of the samples were analyzed using the linear discriminant analysis effect size (LEfSe) method. The results showed the enrichment bacteria *Gluconobacter*, *Providencia*, and *Enterobacteriaceae* in the 30-day-old flies compared to the 10-day-old flies in the control group (Figure 5C). An important follow-up question is whether the microbial diversity affects ROS accumulation processes. We correlated the age-altered family and genus of the gut microbiota with the age-related ROS accumulation module using a Mantel test. The richness of *Gluconobacter*, *Providencia,* and *Enterobacteriaceae* had significant positive correlations with the ROS accumulation module (Figure 5D, *p* < 0.05), which is consistent with previous studies [18,19]. We further evaluated the biological contributions of *Gluconobacter*, *Providencia,* and *Enterobacteriaceae* to ROS accumulation using random forest (RF) analysis and found that *Gluconobacter*, *Providencia,* and *Enterobacteriaceae* are the main variables for predicting ROS accumulation in flies (Figure 5E, R^2^ = 0.813, *p* < 0.001).

To confirm the inhibitory effect of EPs on *Gluconobacter*, *Providencia,* and *Enterobacteriaceae* in the guts of aged flies, we evaluated the relative abundances of *Gluconobacter*, *Providencia,* and *Enterobacteriaceae* in the 10-day- and 30-day-old flies in the control group and the 30-day-old flies in the EP groups. In agreement with previous results, we found that the abundances of *Gluconobacter* (Figure 5F), *Providencia* (Figure 5G), and *Enterobacteriaceae* (Figure 5H) increased significantly in 30-day-old flies compared with 10-day-old flies in the control group, while they were suppressed after EP supplementation. These results indicated that EPs rescue the age-induced structural changes in the gut microbiota of flies.

### 2.6. Association between the Life-Extending Effect and Gut Microbiota of EPs in Drosophila

**Figure 6:**
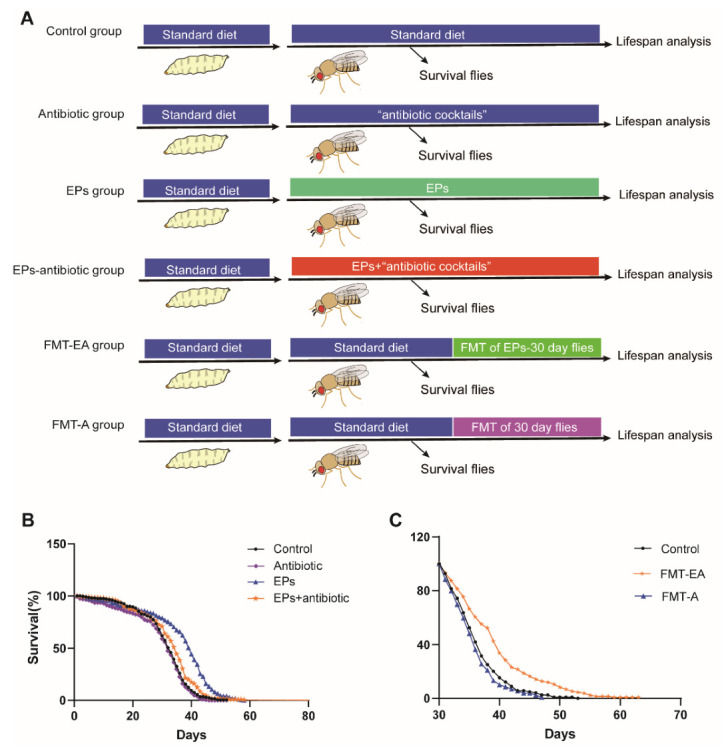
Life-extending effect of EPs achieved via gut microbiota remodeling. (**A**) Outline of the experimental strategy. (**B**) Percentage survival of the control group, antibiotic group, EP+antibiotic group, and EP groups. (**C**) Percentage survival of the control group, FMT-EA group (cultured on the dirty medium in which the 30-day-old flies in EP groups were cultured for 3 days), and FMT-A group (cultured on the dirty medium in which the 30-day-old flies in the control group were cultured for 3 days). Mean ± SEM (*n* = 200). The details of the statistical tests used in this figure are given in the Section 4.

To identify the relationship between life-extending and gut microbiota remodeling effects of EPs on flies, we collected flies after eclosion within 24 h and performed experiments as follows (Figure 6A). In the EP groups, the flies were cultured on a medium supplemented with EPs. In the antibiotic group, the flies were cultured on a medium supplemented with antibiotic cocktails (200 μg/mL of rifamycin, 50 μg/mL of tetracycline, and 500 μg/mL of ampicillin). In the EP+antibiotic group, the flies were cultured on a medium supplemented with both EPs and antibiotic cocktails. In the FMT-EA (fecal microbial transplantation (FMT) of the old flies in the EP groups) group, the flies were cultured on standard medium for 30 days and then transferred to the “dirty medium” (the 30-day-old flies in the EP groups were cultured on the medium for 3 days). In the FMT-A (fecal microbial transplantation (FMT) of old flies in the control group) group, the flies were cultured on standard medium for 30 days and then transferred to the dirty medium (30-day-old flies in the control group were cultured on the medium for 3 days). A separate group of flies was cultured on standard culture medium as the control group. The live flies were counted every day until the last fly died. As previously described, the average life span of flies in the EP groups significantly increased compared to the control group (*p* < 0.01). Nevertheless, the life span of the flies in the EP+antibiotic group was only mildly impacted (Figure 6B and Table 3 ), while the antibiotic group was not affected compared with flies in the control group. In addition, comparison between the FMT-EA group and the control group showed that the FMT of aged flies in the EP groups can also extend the life span of flies significantly, while the FMT of aged flies in the control group mildly shortened the life span of the flies compared to the control group (Figure 6C and Table 3) (*p* < 0.01). These results further confirmed that the life-extending effect of EPs is achieved by gut microbiota remodeling. 

### 2.7. FMT of the 30-Day-Old Flies from the EP Groups has a Similar Effect on the Gut Microbiota as EPs

**Figure 7:**
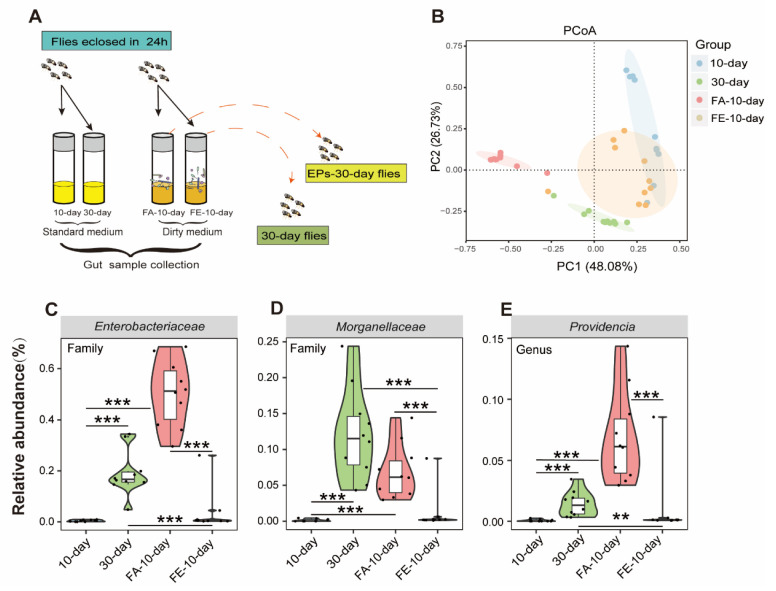
FMT from old flies fed with or without EPs differently affected the gut microbiota in young flies. (**A**) Schematic diagram of the experimental procedure. (**B**) ASV-based PCoA with Bray–Curtis distance (for principal component 1 and principal component 2) showed variations in the gut microbial beta diversity of 10-day-old, 30-day-old, FA-10-day (flies cultured on the dirty medium from 30-day-old flies in the control group for 10 days), and FE-10-day (flies cultured on the dirty medium from 30-day-old flies in the EP groups for 10 days) and assessed with PERMANOVA. (**C–E**) Relative abundance comparison of Gluconobacter (**C**), Providencia (**D**), and Enterobacteriaceae (**E**) in the gut microbiota of 10-day-old, 30-day-old, FA-10-day, and FE-10-day flies detected with 16S sequencing analysis. ** *p* < 0.01, *** *p* < 0.001.

The gut microbiota is a key regulator of host health, especially in aging [20]. We confirmed the relationship between life-extending and gut microbiota remodeling effects. To determine whether the FMT from the 30-day-old flies in the EP groups had a similar effect on the microbiota of flies as EPs, we performed experiments as follows (Figure 7A). 

We analyzed the gut microbiomes of 10-day- and 30-day-old flies as baseline, while the FA-10-day flies (flies were cultivated on the dirty medium from the 30-day-old flies in the control group for 10 days) and FE-10-day flies (flies were cultivated on the dirty medium of the 30-day-old flies in the EP groups for 10 days) as experimental data using 16S rRNA amplicon sequencing. The composition and diversity of the gut microbiota from these flies were assessed. We found clear differences between the 10-day- and 30-day-old flies in the control group in beta diversity, as previously described. However, the microbiota composition of the FE-10-day flies was closer to that of the 10-day-old flies compared to the FA-10-day flies, while the microbiota composition of the FA-10-day flies was closer to that of the 30-day-old flies compared to the FE-10-day flies (Figure 7B). These results indicated that the FMT of the 30-day-flies from the EP groups had a similar effect on the microbiota as EPs. In this study, *Gluconobacter*, *Providencia*, and *Enterobacteriaceae* were proved to be the key target bacteria of EPs. 

We further examined the relative abundances of *Gluconobacter*, *Providencia,* and *Enterobacteriaceae* in 10-day-old, 30-day-old, FA-10-day, and FE-10-day flies. We found that the abundances of *Gluconobacter*, *Providencia,* and *Enterobacteriaceae* considerably increased in 30-day-old and FA-10-day flies and significantly decreased in FE-10-day flies (Figure 7C–E). These results further indicated that EPs rebuild aging-induced structural changes in the gut microbiota of flies and the FMT from the 30-day-old flies in the EP groups has a similar effect on the microbiota as EPs. 

## 3. Discussion

As a traditional Chinese medicine (TCM), Eucommiae cortex display various biological functions, including effects against oxidants, fatigue, and immunomodulation [21]. As one type of the main biological macromolecules in Eucommiae cortex, EPs have exhibited anti-inflammatory activity in previous pharmacological experiments [21]. In this study, by integrating data on the gut microbiota, proteome, and life span, we demonstrated that EP supplementation extends the life span and selectively inhibits *Gluconobacter*, *Providencia*, and *Enterobacteriaceae* in the microbiome in Drosophila during aging. Besides, EP supplementation also ameliorates age-related ROS accumulation, which may also contribute to the prolonged life span effect in flies. 

Various theories have been proposed to explain the progressive decline in cellular function and organismal fitness associated with aging. Free radicals, reactive metabolites, or ROS are classified as oxidative stress when they produce more free radicals than are eliminated by protective mechanisms, including antioxidants. As a result of this imbalance, important biomolecules and cells are damaged, possibly resulting in adverse effects on the entire body [22]. In response to changes in intra- and extracellular environments, reactive oxygen species produced by normal cellular metabolism can stimulate signaling pathways in animal cells [23]. However, when the level of ROS exceeds the cellular antioxidant capacity, a chain of reactions can be triggered, resulting in widespread oxidation [24]. As a result, these reactive species cause uncontrolled reactions with non-target macromolecules (DNA, proteins, and lipids), causing severe DNA damage [25]. Keap1–Nrf2 pathways are responsible for regulating many antioxidant genes that sustain cellular homeostasis in response to oxidative stress [26]. Increasing evidence suggests that polysaccharides show strong antioxidant effects due to broad polarities, complex structures, and large molecular weights [27,28,29]. In previous pharmacological experiments, EPs have been shown to have immunoregulatory, anti-inflammatory, and neuroprotective activities [10,11,30,31]. However, the effects of EPs on age-induced oxidative stress and the Keap1–Nrf2 pathway have not been investigated yet. In this study, we observed that EP supplementation prolongs the life span; reduces age-related ROS accumulation; increases the activity of antioxidant enzymes, such as T-SOD and GSH-Px; and upregulates the Keap1–Nrf2 pathway signal (Figure 4) in Drosophila. Therefore, EP supplementation can effectively mitigate age-related oxidative stress, thus prolonging the life span of Drosophila. 

In mammals and flies, gut microbes play a critical role in gut function and physiology [32]. Studies have shown that aging is associated with changes in the composition of the gut microbiota, inflammation, and increased gut permeability [1,33]. It has been demonstrated that polysaccharides have beneficial effects on the gut microbiota, particularly by reducing pathogen abundance and increasing beneficial bacteria, which contributes to the health of the host [34]. For instance, Arabinoxylan boosts the growth of *Bifidobacterium*, *Lactobacillus*, and *Bacteroides*, while reducing the abundances of *Fusobacterium*, *Bilophila*, and *Desulfovibrio* [35]. Our previous study revealed that EP supplementation inhibits Escherichia coli expansion and reduces lipopolysaccharides (LPS) in serum and colonic contents, inhibiting subsequent neuroinflammation [11]. *Gluconobacter*, *Providencia*, and *Enterobacteriaceae* are ubiquitous opportunistic pathogens of the Gram-negative bacteria of Drosophila [18,36]. In this study, we found high amounts of *Gluconobacter*, *Providencia*, and *Enterobacteriaceae* in aged flies (Figure 5C). Moreover, the abundances of *Gluconobacter*, *Providencia,* and *Enterobacteriaceae* were positively correlated with the ROS accumulation module, as reported in various studies [37,38,39,40]. It is surprising that EPs mitigated age-induced oxidative stress by inhibiting the expansion of *Gluconobacter*, *Providencia*, and *Enterobacteriaceae* in the guts of Drosophila (Figure 5). The viewpoint was verified in the FMT and microbe-clearing experiments (Figure 6 and Figure 7). Therefore, we believe that the life-prolonging effect of EPs in Drosophila is primarily explained by remodeling gut dysbiosis. 

In summary, our study of Drosophila illustrated that individual ROS accumulation is caused by gut dysbiosis and upregulation of Keap1–Nrf2 pathways in response to aging. These results contribute to a more in-depth understanding of aging mechanisms. The microbiota can be influenced by the environment, such as diet, which influences the physiology, metabolism, and behavior of adults [41]. Notably, EPs can remodel the gut microbial composition, relieve ROS accumulation, and prolong the life span of Drosophila. In addition, EPs effectively improve age-induced declines, such as locomotor activity and fecundity. These findings provide new perspectives on age-related disorders and healthy aging. Thus, EPs can be used as prebiotics to remodel the age-related gut microbiota composition and deal with age-related dysfunction.

## 4. Materials and Methods

### 4.1. Preparation of EPs

We purchased Eucommiae *cortex* from the Puzheng Pharmaceutical Company (Nanchang, China). The extraction and purification of EPs were performed as follows:

Crude polysaccharides were extracted using hot water extraction (100 °C, three times boiled). Next, three-phase partitioning was carried out by adding (NH4)2SO4 (25% *w*/*v*) to the crude polysaccharides. HCl and NaOH were added to the mixture to adjust the pH to 6, and then, *tert*-butanol at a mixture: *tert*-butanol ratio of 1:2 was added. The mixture was left to stand for 30 min (30 °C) and then centrifuged (2000 r/min, 20 min). Finally, the lower phase was freeze-dried to obtain crude EPs [42]. The EPs were purified using a DEAE-52 cellulose column and eluted with distilled water and gradient NaCl solutions. The eluted fractions were pooled and concentrated, and the concentration of neutral saccharides was determined using the phenol-sulfuric acid method. The polysaccharide fractions were again dialyzed against water for 3 days and lyophilized [43].

### 4.2. Characterization of EPs

The analyses of EPs, including the analysis of the monosaccharide composition and molecular weight, and scanning electron microscopy (SEM) were performed by the Beijing ZKGX Research Institute of Chemical Technology (Beijing, China), as described before [11].

### 4.3. Drosophila Stocks and Culture

Drosophila melanogaster were cultured on standard yeast–cornmeal–sucrose medium (720 mL of H_2_O incorporated with 60 g of corn flour, 48 g of sucrose, 9 g of dry yeast, 6 g of agar power, and 2.4 mL of propionic acid for each medium) in a temperature- and humidity-controlled incubator with a 12 h/12 h light/dark cycle at 25 °C. Flies eclosed within 48 h were collected under brief CO_2_ anesthesia, housed at a density of 20 flies (10 females and 10 males) per vial, and transferred to fresh culture medium every 3 days during the experiment. 

### 4.4. EPs Supplementation and Antibiotic Treatments

For the EP supplementation groups, EPs were added to the medium to obtain the following final concentrations of 1%, 2%, 4%, 8%, and 16%. To eliminate the gut microbiota in the antibiotic group, flies were transferred to a diet containing “antibiotic cocktails” (a combination of 200 mg/mL of rifamycin, 50 mg/mL of tetracycline, and 500 mg/mL of ampicillin) [44]. In the EP+antibiotic group, flies were transferred to an EP supplementary diet containing antibiotic cocktails after eclosion and maintained on an EP+antibiotic diet continuously for survival analysis.

### 4.5. Assays of Life Span, Body Weight, and Egg-Laying

Flies of the same age were collected and reared at a low density of 20 flies (10 females and 10 males) per vial and 10 vials for each group at 25 °C. For life span assay, we counted the live flies every day during the experiment and transferred them to new vials every third day for body weight measurement, and 10 adult flies were together weighed (10 females or 10 males per sample) using an ultrasensitive scale (AUY220, SHIMADZU, Kyoto, Japan). For egg-laying assay, single females were transferred to a fresh tube and eggs were counted every day. For these assays, 10 replicates were performed in each group. 

### 4.6. Locomotor Activity

Locomotor activity was assessed using elongated vials. A mark line was drawn at 8 cm from the bottom of each vial. We transferred 20 flies (10 males and 10 females) to the elongated vials and allowed them to acclimatize for 10 min. Next, the vials were tapped three times with proper strength to ensure that all flies fell to the bottom. The number of flies that crossed the mark line within 15 s were recorded. Ten replicates were performed in each group.

### 4.7. Assays of Antioxidant Enzyme Activity

Superoxide dismutase (SOD) and glutathione peroxidase (GSH-Px) in flies were measured using SOD and GSH-Px assay kits (Nanjing Jiancheng Bioengineering Institute, Nanjing, China) in accordance with the instructions of the manufacturer. The absorbance of GSH-Px and SOD at 550 and 412 nm was measured to evaluate their activity. Finally, antioxidant enzyme activity was normalized to each protein concentration.

### 4.8. Midgut and Hindgut Dissection

Flies were flushed with 75% alcohol after CO_2_ anesthesia and then washed extensively with 1×phosphate buffered saline (PBS) before dissection. The midguts were dissected in cold PBS by carefully removing the tracheae, Malpighian tubules, and crops. Each sample for microbiota analysis contained at least 30 midguts (15 females and 15 males), and 9–10 biological replicates were performed in each group. 

### 4.9. ROS Measurement

In situ ROS detection was performed using dihydroethidium (DHE) staining (Solarbio, Beijing, China) following previously described protocols [45,46]. For DHE staining, flies were anesthetized with CO_2_ and dissected in cold PBS under a dissecting microscope. In ROS staining assay, 30 guts and 8 brains were assayed. The dissected brains and guts were incubated at 25 ℃ with 60 mM DHE for 25 and 15 min, respectively. Next, we washed the samples for 3 × 5 min in PBS at room temperature. Before mounting, the samples were incubated at 25 °C with 4’,6-diamidino-2-phenylindole (DAPI) for 10 min and then washed three times for 5 min with PBS at room temperature. All steps were performed on a shaker in the dark. We mounted the samples in each independent experiment between glass slides and coverslips using Fluoromount G (Southern Biotech, Birmingham, AL, USA) and immediately imaged them with a 10×objective on a Leica SP8 confocal microscope (TCS SP8, Leica, Wetzlar, Germany).

### 4.10. DNA Extraction and PCR Amplification and Sequencing

Homogenized samples were stored at −80 °C, and gut DNA was isolated using a QIAamp DNA Stool Mini Kit (QIAGEN, Hilden, Germany). We checked the DNA quality on 1% agarose gels and amplified the V4 regions of 16S rRNA genes using barcoded primers 515F (5′-GTGCCAGCMGCCGCGGTAA-3′ and 806A (5′-GGACTACHVGG GTWTCTAAT-3′). Sequencing steps were carried out by the Magi Gene Technology Company (Guangzhou, China).

### 4.11. Sequencing and Analysis of 16S rRNA

Illumina Miseq PE250 was used to generate raw sequence data of bacterial 16S rRNA genes. Further analysis of the sequences was performed on the QIIIME2 platform (version 2019.10). Principal coordinate analysis (PCoA) was performed using unweighted data. The Phyloseq package was used to apply Divisive Amplicon Denoising Algorithm 2 (DADA2) to these sequences, generating tabular representations of representative sequences and amplicon sequence variants (ASVs) [47]. We used the RDP Bayes-Classifier with an 80% confidence threshold to classify the taxonomy of each representative 16S bacterial gene sequence [48]. All 16s rRNA data sets are publicly available (GEO: GSE98944 and GSE98945).

### 4.12. Mass Spectrometry Measurement and Quantitative Proteomic Analysis

The analysis was performed as previously described [49,50]. Samples of 50 flies (25 males and 25 females) were lysed in ice-cold radioimmunoprecipitation assay (RIPA) buffer (Solarbio, Beijing, China) with 1 mM phenylmethylsulfonyl fluoride (PMSF; Solarbio, Beijing, China) in it. Specifically, 20 mg of tissues was lysed with 200 µL of lysis buffer in an oscillating grinder at 4 °C, with the addition of 2 steel beads. The lysate was further centrifuged at 3600 rpm for 10 min at 4 °C to remove the remaining cuticle fragments. The protein concentration was analyzed using the Pierce™ BCA Protein Assay (Solarbio, Beijing, China) according to the instructions of the manufacturer. Three biological replicates were performed in each group.

Protein samples were digested with trypsin at 37 °C for 14 h, and the obtained peptides in each group were resuspended in 200 µL of 0.1% formic acid (0.1% FA) and desalted using the C18 stage tip method described previously [51]. The peptides were vacuum-dried and stored at −20 °C until LC-MS analysis using ultra-high-resolution LC-MS (Thermo Fisher Scientific, Waltham, MA, USA). MaxQuant (version 2.0.3.0) was used to search the tandem mass spectra against the unreviewed Drosophila melanogaster UniProt database (version: 202200208, 39,699 sequences). A label-free quantification algorithm was used in MaxQuant. We used proteins with at least two identifications in each group for the subsequent quantitative analysis. Missing values were imputed with the minimum value in the proteome data set for algorithms in which missing values cannot be handled. All the experiments were performed in triplicate (*n* = 3). To compare the means of treated and control groups, we conducted one-way ANOVA in GraphPad Prism along with a multiple comparison analysis using the Tukey test.

### 4.13. Statistical Analysis

For all Kaplan–Meier survival curve analyses, statistical analysis was performed using OASIS_2_ [52], and other statistical analyses were performed using GraphPad Prism 9X. In addition, a two-tailed Student’s t-test was used for further comparisons between the two samples, and one-way analysis of variance with Tukey’s test was used for multiple comparisons. 

## Figures and Tables

**Table 1:** The monosaccharide composition of EPs.

Monosaccharide	(% *w/w*)
Mannose	1.12872
Rhamnose	2.18416
Galacturonic acid	3.15037
Glucose	82.70771
Galactose	0.79398
Xylose	2.01605
Arabinose	1.99197

**Table 2:** Statistics of survival curves in Figure 2B–G. Cohort sizes, mean and median life spans, percentage changes, and log-rank tests of the Kaplan–Meier survival curves in this study.

Figure 2B–G	*n*	Mean (% Change)	Median (% Change)	Log-Rank (vs. Control)
Control	200	30.41	32	-
1% EPs	200	31.37 (+3.2%)	32 (+0)	*p* > 0.05
2% EPs	200	33.02 (+8.6%)	34 (+6.3%)	*p* < 0.01
4% EPs	200	34.81 (+14.5%)	38 (+18.8%)	*p* < 0.01
8% EPs	200	26.11 (−14.1%)	27 (−15.6%)	*p* < 0.01
16% EPs	200	21.43 (−29.5%)	21 (−34.4%)	*p* < 0.01

**Table 3:** Statistics of survival curves in Figure 6. Cohort sizes, mean and median life spans, percentage changes, and log-rank tests of the Kaplan–Meier survival curves in this study.

Figure 6B,C	*n*	Mean (% Change)	Median (% Change)	Log-Rank (vs. Control)
Control	200	30.41	32	-
4% EPs	200	34.81 (+14.5%)	38.00 (+18.8%)	*p* < 0.01
Antibiotic	200	29.67 (−0.7%)	32.00	*p* > 0.05
EP+antibiotic	200	31.85 (+4.5%)	34.00 (+6.2%)	*p* < 0.01
FMT-EA	200	32.81 (+7.3%)	34.00 (+6.2%)	*p* < 0.01
FMT-A	200	29.15 (−1.3%)	33.00 (1%)	*p* > 0.05

## Data Availability

DATA is contained within the article.
